# Association of Human Papilloma Virus and Epstein-Barr Virus with Ovarian Cancer in Shiraz, Southwestern Iran

**DOI:** 10.30699/ijp.2020.119681.2306

**Published:** 2020-07-16

**Authors:** Mohammad Reza Shokouh, Akbar Safaei, Afagh Moattari, Jamal Sarvari

**Affiliations:** 1 Department of Bacteriology and Virology, Shiraz University of Medical Sciences, Shiraz, Iran; 2 Department of Molecular Pathology, Shiraz University of Medical Sciences, Shiraz, Iran; 3 Gastroenterohepatology Research Center, Shiraz University of Medical Sciences, Shiraz, Iran

**Keywords:** Epstein-Barr virus, Human Papillomavirus, Ovarian Cancer

## Abstract

**Background & Objective:**

Ovarian cancer is one of the most common cancers amongst women. The association of Human papillomavirus (HPV) and Epstein-Barr virus (EBV) with ovarian cancer is inconclusive; therefore, the aims of this study were to evaluate the frequency of HPV and EBV in malignant, borderline, benign and normal ovarian tissues.

**Methods:**

In this case-control study, 205 Paraffin-embedded ovarian tissue specimens including 68 malignant, 27 borderline, 65 benign, and 45 normal tissues were included from December 2014 to January 2018 and subjected to DNA extraction. The β-globin gene was amplified using PCR to confirm the quality of the extracted DNA. The genomes of HPV (genotypes 16 and 18) and EBV were identified, using specific primers by PCR.

**Results:**

The mean age of participants was 43.42 ± 15.4 years. The frequency of HPV was statistically significant between malignant versus benign (*P*=0.02) and control groups (*P*=0.002), but not with borderline tumor group (*P*=0.78). Amongst HPV infected samples, 1 (4.5%) and 14 (63.6%) samples were infected with types 16 and 18, respectively. Also 4 (18.2 %) samples were infected with both genotypes. Eleven samples including 7(10.3%) malignant, 1 (3.7%) borderline, 3 (4.6%) benign and none (0%) of normal control groups were infected with EBV, which was statistically different between malignant and the normal control group (*P*=0.03).

**Conclusion:**

The results of our study showed the possible role of high risk HPVs as well as EBV in pathogenesis of ovarian cancer, and further studies are recommended to confirm these findings.

## Introduction

Ovarian cancer is the eighth most common cancer amongst women (1). There were nearly 300,000 new cases in 2018 (1). The World health organization estimated a 55% increase in the incidence as well as mortality of ovarian cancer till 2035. Ovarian Cancer has registered as the 24^th^ and 19^th^ death related disease in Iran and the world, respectively (1,2). 

The exact causes of ovarian cancer remain unclear; however, a number of risk factors, such as age, alcohol consumption, smoking, stress, family history, early menstruation, late menopause and infertility have been supposed to be linked to this type of cancer development (3). According to previous studies, viral infections including Human Papillomavirus (HPV), Epstein-Barr virus (EBV), Hepatitis B virus (HBV) and Hepatitis C virus (HCV) have been responsible for 15-20% of cancers (4,5). 

HPV infection is limited to the basal cells of stratified epithelium, the only tissue in which they replicate (6). The major oncoproteins including E6 and E7 of high risk HPVs induce cell transformation through inactivation of two cellular tumor suppressor proteins, p53 and pRb, respectively (7). Inactivation of pRb by E7 bypasses the cell limitations to enter S phase in the infected cells whereas protosomal degradation of p53 by E6 ensures cell survival by preventing apoptosis. Moreover, viral genome integration into host DNA genome enhance the expression of E6 and E7 that leads to cellular proliferation and malignancy (7). 

Although the association of HPV with cervical cancer is well defined (8), the role of HPV in other cancers, such as esophageal squamous cell carcinoma, lung, prostate, breast, skin, colorectal, urinary tract and ovarian cancers has not been proven conclusively (9-11). In this regards, in Iran, Farzane *et al. *could not find any association between HPV infection and ovarian cancer (12), but Dadashi* et al. *suggested that HPV infection is associated with ovarian cancer (13). 

EBV is the first human virus to be assigned as carcinogenic pathogen. It belongs to the *Herpesviridae* family and estimated to be among the most common viruses in humans (14). Some EBV encoded proteins including BZLF1 and LMP (late membrane protein) related molecules are involved in the carcinogenesis process. BZLF1 induces matrix metallopeptidase 9, thereby complexes with P53 and P56 to prevent the apoptosis. Moreover, BRL1 induces E2F release and subsequently entering the infected cells into S-phase (15). In addition, LMP-1 and LMP-2 continuously activate several growth signaling pathways that result in the proliferation of the infected cells. Furthermore, EBV encodes several un-translating transcripts such as EBERs, BARTs and viRNA that perform some roles in immune evasion and cell survival (16).

Regarding the association of several cancers with HPV and EBV as well as the published data regarding the association of these viruses with ovarian cancer, this study was conducted to determine the frequency of HPV and EBV in malignant, borderline, benign, and normal ovarian tissues in Shiraz, Iran.

##  Materials and Methods

**Fig 1:**
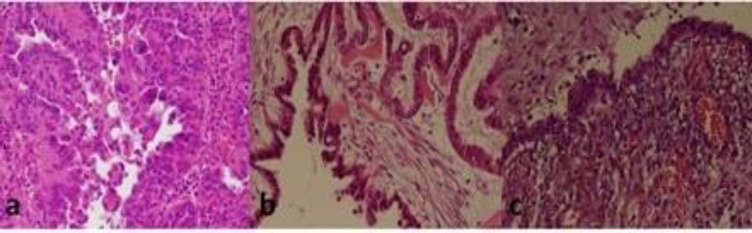
Photomicrograph of human ovarian tumor tissue**. A)** A photomicrograph of human ovarian serous cyst adenocarcinoma infected with HPV DNA. **B)** A photomicrograph of human ovarian serous cyst tumor borderline infected with HPV DNA. C) A photomicrograph of human ovarian endometrium benign lesion infected with HPV DNA. (H and E×250).

From December 2014 to January 2018, 205 paraffin-embedded biopsy specimens that were histologically confirmed as malignant, borderline, benign and normal ovarian tissue were included consecutively in this case-control study (Figure 1). Samples were collected from Faghihi hospital, affiliated with Shiraz University of Medical Sciences (SUMS). The study was approved by the local Ethics Committee of SUMS (SUMS. 1396-15664). 

**DNA Extraction and Qualification**

Deparaffinization step of paraffin-embedded biopsy specimens was performed as previously described (17). Briefly, 15 sections (with thickness of 30 μm) of paraffin-embedded block were deparaffinized using 1200 μL xylene. The tube was thoroughly vortexed, incubated for 30 min at room temperature and then underwent centrifugation (14000 rpm). After that, the supernatant was removed, 1200 μL absolute ethanol was added and the mix was incubated at room temperature for 5 min and then underwent centrifugation (14000 rpm) to remove the supernatant. Both steps were repeated once more. Finally, the tubes were incubated at 37°C on a heating block until ethanol was totally evaporated. The DNA was then extracted, using a Tissue Genomic DNA Extraction Mini Kit (Yektatajhiz Inc., Tehran, Iran) according to the manufacturer’s instruction. The extracted DNA was stored at -20°C until further use.

The extracted DNA was initially subjected to PCR with consensus primers PCO3/PCO4 targeting β-globin to ensure the quality of samples (Table 1). Negative samples were excluded from next steps of the study. For this purpose, the PCR reaction was performed in a total volume of 25 μL as it was previously described (17).

**HPV Genome Detection**

HPV genome detection was performed on extracted DNA samples with positive results for β-globin gene as described previously (17). Briefly, using two separate sets of HPV specific primers, L1 region of the virus was targeted and amplified by a nested PCR reaction (Table 1). The program in the first round was adjusted as follows; 5 min initial denaturation at 95°C, 40 cycles of denaturation at 95°C for 45 s, annealing at 55°C for 55 s, extension at 72°C for 60 s, and one step of final extension at 72°C for 10 min. The program in the second round was adjusted as follows: 5 min initial denaturation at 95°C, 40 cycles of denaturation at 95°C for 45 s, annealing at 55°C for 40 s, extension at 72°C for 45 s, and one step of final extension at 72°C for 10 min. PCR products were then loaded into 1.5% agarose gel and visualized under UV light.

**High Risk HPVs Genotyping**

To determine high risk HPV16 and 18 genotypes among the positive samples, we performed two sets of PCR, using HPV16 and 18 specific primers which amplify E6 region of a specific genotype as previously described (18) (Table 1). PCR was performed in a total volume of 15μL, containing MgCl2 0.75 μL (CinnaGene, Iran), dNTPs 0.5 μL (CinnaGene, Iran), reaction buffer 2.5 μL (CinnaGene, Iran), Taq DNA polymerase 0.25 μL (CinnaGene, Iran) ,0.5 μL each specific primers (F and R16/F and R18), water 8 μL and DNA template 2 μL. PCR program for HPV genotyping was adjusted as follows: 5 min initial denaturation at 94°C, 40 cycles of denaturation at 94°C for 1 min, annealing at 51°C for 1 min, extension at 72°C for 1 minutes, and final extension at 72°C for 5 min. PCR products underwent electrophoreses on 1.5% agarose gel and visualized under UV light.

**Table 1:** The Sequences and other Characteristics of Primers Used in this Study

*References*	*Size, bp*	*5*'*to 3*'* Sequence*	*Primers*	*Locus*
	110	5'- ACACAACTGTGTTCACTAGC-3'	PCO3	β-globin
		5'- CAACTTCATCCACGTTCACC-3'	PCO4	
	450	5'- CGTCCMARRGGAWACTGATC-3'	MY09	HPV L1
		5'-GCMCAGGGWCATAAYAATGG-3'	MY11	
	149	5'-TTTGTTACTGTGGTAGATACTAC-3'	GP5+	
		5'-GAAAAATAAACTGTAAATCATATTC-3'	GP+6	
	141	5'-AGGAGGATGAAATAGATGG-3'	16F	HPV E6
		5'-CTTCCAAAGTACGAATGTC-3'	16R	
	92	5'-CGATGAAATAGATGGAGTTAA-3'	18F	
		5'-CACACTTACAACACATACA-3'	18R	
		5′- TACTCCTTACTATGTTGTG-3′	EBV F	EBV BZLF1
	295	5′-CCTTGCCTAATATCCTAC-3′	EBV R	

**EBV Genome Detection**

EBV genome amplification was performed on samples with positive results for β-globin gene as previously described (19). PCR was performed in a total volume of 12μL, containing MgCl2 0.75 μL (CinnaGene, Iran), dNTPs 0.5 μL (CinnaGene, Iran), reaction buffer 2.5 μL (CinnaGene, Iran), Taq DNA polymerase 0.25 μL (CinnaGene, Iran) ,0.5 μL each specific primers (Table 1), water 5 μL and DNA template 2 μL. Briefly, PCR program for amplification of BHRF1 (BamH leftward reading frame-1) region of the EBV genome was as follows: 10 min initial denaturation at 95°C, 40 cycles of denaturation at 95°C for 30 s, annealing at 57.6°C for 45 s, extension at 72°C for 45 sec following a final extension step at 72°C for 10 min. PCR products were electrophoresed in 1.5% agarose gel and visualized under UV light.

**Statistical Analysis**

Statistical analysis was performed using SPSS 23 (SPSS Inc., Chicago, IL, USA) for Microsoft Windows®. The results were processed statistically using the Chi-square test and t-test. P-value less than 0.05 was considered as statistically significant.

## Results

**Demographic and Pathological Findings**

In this study, a total of 205 ovarian tissue samples were divided into 4 groups of group 1: 68 (33.2%) malignant, group 2: 27 (13.2 %) borderline, group 3: 65 (31.7%) benign and group 4: 45 (21.9%) ovarian tissue samples that were obtained from individuals without any ovarian disease (ovarian cyst) as the control group. In addition, 68 malignant tumors were also divided into three subgroups of grade I with 31 samples (45.6%), grade II with 14 samples (20.6%) and grade III with 23 samples (33.8%). The histopathology finding of the studied samples is shown in Table 2.

The mean age of all participants was 43.42±15.4 in the age range of 13-80 years. 

The mean ages of patients with malignant, borderline, benign and normal controls were 53.18±12.4, 42.83±15.3, 42.19±13.7 and 35.27±12.5, respectively, which was significantly different between malignant and the control groups (*P*=0.043). 

**Table 2:** The 1.5% Gel Electrophoresis of HPV type 16 and 18 PCR products, using type-specific primers. C+: positive control; C–: Negative control; Lanes: 1 and 2 (a positive sample).

Total	Benign	Borderline	Malignant	Histopathology
113	49	17	47	**Serous adenocarcinoma**
34	16	7	11	**Mucinous adenocarcinoma**
8	0	2	6	**Endometrioid adenocarcinoma**
5	0	1	4	**Clear Cell carcinoma**
160	65	27	68	**Total**

**PCR for HPV**

The results showed that of 205 samples, 22 (10.8%) had positive results for HPV. In detail, 13 (19.1%) samples of malignant group (3 with grade I, 3 with grade II and 7 with grade III), 6 (22.2%) samples of borderline tumor, 3 (4.6%) samples of benign group and none (0%) of the control group had positive result for HPV. Statistical analysis showed that the frequency of HPV in malignant group was higher than benign (*P*=0.02) and control groups (*P*=0.002). Moreover, the frequency of HPV was statistically different between borderline tumor versus benign (*P*=0.03) and the control groups (*P*=0.004). Moreover, the frequency of HPV was higher in benign group than control group, but it was not statistically significant (*P*=0.21) (Table 3).

**HPV Genotyping**

The results of HPV genotyping showed that of 22 samples with positive results for HPV, 1 and 14 samples were infected with HPV genotype 16 and 18, respectively. Four (18.2%) samples were infected with both genotypes. Genotypes in three (13.6%) HPV positive samples were not identified. All samples that were infected with HPV type 16 were from patients with malignant tumors (1 case grade I, 1 case grade II and 3 case grades III). HPV genotyping also showed that 18 samples including 12 samples of malignant tumor (3 cases grade I, 2 cases grade II and 7 cases grade III), 4 samples of borderline tumor and 2 samples of benign tumor have been infected with HPV type 18. An 80-year-old participant with grade III malignant tumor was infected with HPV types 16, 18, and also EBV (Table 3). 

**The Detection of EBV Genome**

The PCR results on tissue groups showed that of 205 samples, 11(5.4%) including 7 malignant, 1 borderline and 3 benign samples had positive results for EBV genome. The frequency of EBV was significantly higher in malignant group than the control group (*P*=0.038). In detail, 2 cases with grade I malignancy, 5 cases with grade III malignancy and 1 case with borderline malignancy as well as 3 samples from benign group had positive results for EBV genome. Furthermore, none of cases in the control group had a positive result for EBV genome. Moreover, the frequency of EBV in patients with benign tumors was higher than those of the control group, but it was not statistically significant (*P*=0.21) (Table 3).

**Table 3.** The frequency of HPV and EBV in four study groups

**Group**		**Malignant tumor (n=68 )**				**Borderline tumor** **(n=27 )**	**Benign tumor** **(n= 65 )**	**Normal group** **(n=45 )**	P*-*value
		Total	Grade I(n=31 )	Grade II(n=14 )	Grade III(n=23 )				
**HPV**	Total	13 (19.1%)	3 (9.7%)	3 (21.4%)	7 (30.4%)	6 (22.2%)	3 (4.6%)	0 (0%)	0.002
	HPV16	5 (7.4%)	1 (3.2%)	1 (7.1%)	3 (13%)	0 (0%)	0 (0%)	0 (0%)	
	HPV18	12 (17.6%)	3 (9.7%)	2 (14.3%)	7 (30.4%)	4 (14.8%)	2 (3.1%)	0 (0%)	
	HPV 16/18	4 (5.9%)	1 (3.2%)	1 (7.1%)	2 (8.7%)	0 (0%)	0 (0%)	0 (0%)	
**EBV**		7 (10.3%)	2 (6.4%)	0 (0%)	5 (21.7%)	1 (3.7%)	3 (4.6%)	0 (0%)	0.03

## Discussion

The results of the present study showed that the frequency of HPV was significantly higher in malignant than benign and normal control groups, but not than borderline tumor group. Amongst 22 HPV infected samples, 1 and 14 samples were infected with HPV 16 and 18, respectively. Also 4 samples were co-infected with both genotypes. Moreover, these results also demonstrated that the frequency of EBV was significantly different between malignant and the normal control groups. 

Ovarian cancer is the eighth and ninth most common cancer amongst women in the world and Iran, respectively (1,2). Although HPVs have a clear and distinct role in cervical cancer, its role in the development of cancers of upper genital tract such as ovaries is unclear (20,21).

The results of present study showed that the frequency of HPV infection in malignant tumor groups was significantly higher than benign and the control groups. In agreement with our results, Dadashi* et al. *reported that the frequency of HPV in malignant ovarian tissue was significantly higher than the tissues from benign participants (13). Moreover, Al-Shabanah* et al. *reported that 42% and 8% of the ovarian carcinoma samples and normal adjacent tissues as the control group had positive result for HPV, which was statistically significant (22). In addition, several studies investigated the frequency of HPV infection only in malignant ovarian specimens. For example, Hammou* et al. *reported that the frequency of HPV infection was 8.7% in ovarian cancer samples (23). Moreover, Hassan* et al. *and Mahmood* et al. *reported 10% and 9.6% frequency of HPV infection in ovarian cancer tissue in Saudi Arabian and Iraqi patients, respectively (24, 25). Also, in two studies by Atalay* et al. *and by Bilyk* et al. *on ovarian specimens, 8.5% and 16.9%% of the samples had positive results for the presence of HPV, respectively (26, 27). A marked high frequency of HPV infection in malignant ovarian tissue (66.6%) was reported by Li* et al. *in China (28). 

In contrast, several studies reported a very low or even zero HPV frequency in ovarian cancer specimens. For example, Ingerslev* et al. *reported that only 1/195(0.5%) of tissues from patients with ovarian malignancy had positive results for HPV (29). Similarly, in the studies by Idahl* et al. *and Wentzensen* et al. *none of ovarian cancer samples were determined to have positive results for HPV (20,30). In this regard, Farzaneh* et al. *reported that none of the 105 samples from Iranian patients with benign and malignant ovarian tumors had positive results for HPV (12). Study by S. Shanmughapriya and colleagues in India showed no statistical difference between the frequency of HPV in malignant, benign and control groups (31).

In sum, similar to our results, several studies have suggested the association between HPV and ovarian cancer, while some others did not support this finding. This controversy might be due to the frequency of HPV infection in different areas, sexual behavior as well as the detection method sensitivity, sample type (fresh or fixed tissues), and even the possibility of cross contamination. 

Our results showed that of 22 HPV-infected samples, most (63.6%) were infected with genotype 18. Zimna* et al. *determined that all 7 HPV positive samples were genotype 18 (32). Moreover, Zhang* et al. *reported that 25 (7.7%) of their samples were infected with HPV type 18 (33). Furthermore, Ingerslev* et al. *reported that the only HPV positive sample, was infected by genotype 18 (29). On the other hand, Yang* et al. *reported that of 19 HPV positive samples, 18 were infected with type 16, and only 1 sample was infected with type 18 (34). Al-Shabanah* et al. *also reported that the prevalence of HPV type 16 (42.9%) was higher than type 18 (26.2%) in malignant ovarian tissues (22). Hassan* et al. *reported that 50% and 40% of the infected samples were contained HPV type 16 and 18, respectively (24). Mahmood* et al. *and Malisic at el. reported that all HPV positive ovarian cases from Iraq and Serbia were infected with HPV type 16 (25,35).

 In addition, our study showed that 4 (18.2%) samples were co-infected with HPV types 16 and 18. In this regards, Al-Shabanah* et al. *reported that 7 (16.6%) samples were co-infected with these two high risk HPV types (22).

Results of our study also showed that the frequency of EBV in malignant tumor tissues was significantly higher than the control group. In line with this, Ingerslev* et al. *reported that 10/191(5.2%) of ovarian malignant tissues and 1/174(0.5%) of the control group had positive results for EBV DNA, indicating the association between EBV and ovarian cancer (36). Moreover, Pandya* et al. *showed that the expression of miR-BART7 of EBV was significantly higher in cancerous tissues compared to noncancerous ones (37). Furthermore, Littman* et al. *reported that EBV antibody titers and old-age at primary infection with EBV might have a role in the etiology of ovarian cancer (38). Although it is early to conclude the role of EBV in the development of ovarian cancer, the role of this virus in some important human cancers such as, Burkitt lymphoma, nasopharyngeal carcinoma and gastric cancer is well-defined (39).

In our study, there was a significant association between the mean age of patients with malignant ovarian cancer and the control group. Similarly, Al-Shabanah* et al. *and Mahmood* et al. *reported a significant association between the age of patients with ovarian cancer and the control group (22, 25).

## Conclusion

In conclusion, the results of the present study showed that the frequency of HPV in malignant ovarian samples was significantly higher than normal and benign ones and most genotypes were determined to be high risk HPV types 16 and 18. There was also a significant association in the frequency of EBV in malignant ovarian samples compared to the control group. Altogether, it is suggested that HPV and EBV to be involved in the pathogenesis of ovarian cancer. Further studies would be warranted to show a more clear association.
